# A pin in appendix within Amyand's hernia in a six-years-old boy: case report and review of literature

**DOI:** 10.1186/1749-7922-5-14

**Published:** 2010-05-19

**Authors:** Sadik S Llullaku, Nexhmi Sh Hyseni, Baton Z Kelmendi, Hysni J Jashari, Antigona S Hasani

**Affiliations:** 1 Department of pediatric surgery, University Clinical Centre of Kosova, 10 000 Prishtina, Kosova; 2 Department of anesthesiology, University Clinical Centre of Kosova, 10 000 Prishtina, Kosova

## Abstract

**Introduction:**

Presence of vermiform appendix (non-inflamed or inflamed) in inguinal hernia is called Amyand's hernia in honor to surgeon C. Amyand who published the first case of perforated appendicitis within inguinal hernia in a boy caused by ingested pin. This presentation of foreign body Amyand's hernia appendicitis is very rare, and here we present such a case.

**Case presentation:**

A 6-year-old boy, white Kosovar ethnicity, presented with right groin pain, swelling and redness. Two days before admission the patient was injured by football during a children game in the right lower abdomen and the next day he complained of pain in the right inguinal area.

On admission patient had a painful non-reducible mass in the right inguinal region and cellulitis. Plain abdominal x-ray showed no fluid-air levels, but a metallic foreign body (pin) under right superior pubic ramus was apparent. With preoperative diagnosis of suspect incarcerated inguinal hernia with cellulitis the patient was operated on under general anaesthesia in December 2, 2006. Intraoperatively we found the inflamed vermiform appendix perforated by a pin in the hernial sac. Appendectomy and herniotomy were performed. The wound was primary closed, without any post-operative complications and follow up for the patient is three years long.

**Conclusion:**

Foreign body (pin) Amyand's hernia appendicitis seems to be extremely rare, maybe once in a century (Amyand 1735, Hall 1886, and our case in 2006). In patients with clinical signs of incarcerated inguinal hernia, with locally inflammatory signs, but without signs of intestinal obstruction Amyand's hernia appendicitis in differential diagnosis must be considered. In our case, it is possible that the injury during the football game might have induced perforation of the vermiform appendix with the foreign body in it.

## Background

The finding of vermiform appendix in inguinal hernia is called Amyand's hernia. The Amyand's hernia was described in a 11-year-old boy who presented with inflamed appendix in inguinal hernia sac perforated by a pin. In treatment of this case Claudius Amyand in 1735 performed appendiceal resection [1]. He is credited for the first successful appendectomy [2,3]. In his honor inguinal hernia containing vermiform appendix is given his name. Claudius Amyand (1680-1740) a French refugee surgeon was sergeant surgeon to King George II and principal surgeon to the St. George's and the Westminster hospitals of London.

## Case presentation

A 6-year-old boy, weighing 18.5 kg, white Kosova-Albanian ethnicity, presented with right groin pain, swelling and redness. Two days before admission the patient was injured during a football game in the right lower abdomen and the next day he complained of pain in the right inguinal area.

Abdominal pain was permanent and increasing. The child was anorexic, but had no complaints of vomiting and diarrhea or disuria. On admission the patient was sub febrile (38°Celsius) with a painful non-reducible mass in the right inguinal region with signs of cellulitis in this area. There was a marked tenderness on palpation of the right lower abdomen and right hemiscrotum was moderately swollen and painful in palpation.

**Figure 1:**
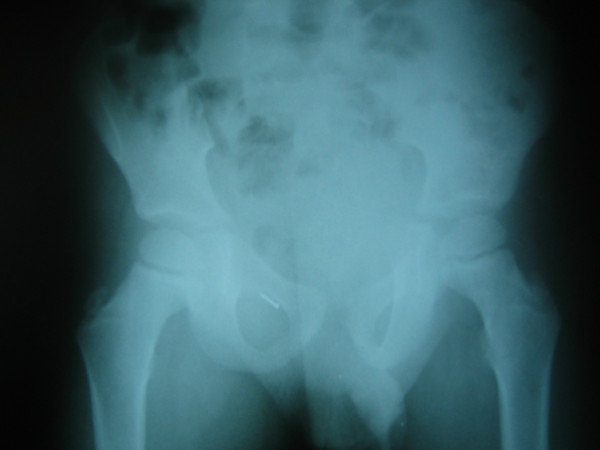
**Preoperative plain abdominal x-ray in erect position**. Metallic foreign body (pin) under right superior pubic ramus is seen. No air-fluid levels suggesting intestinal obstruction are seen.

**Figure 2:**
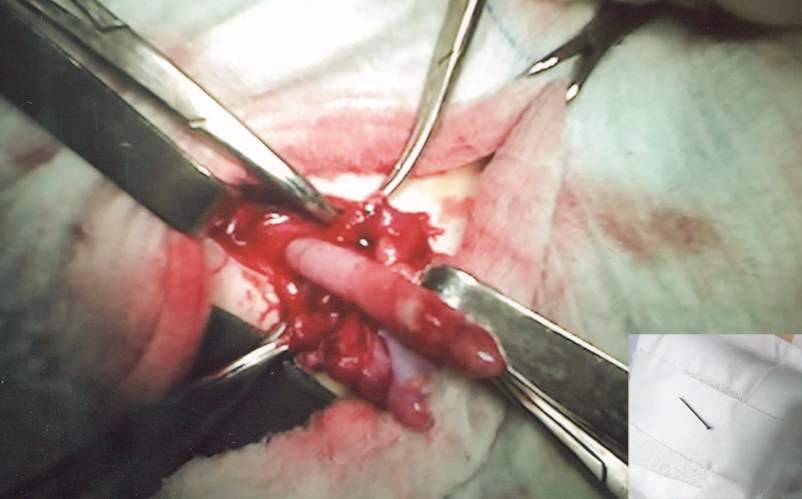
**Inflamed by pin perforated vermiform appendix in hernia sac in right inguinal hernia**. Pin has perforated appendix in distal part, and purulent fluid in the hernia sac was collected. In the corner of the figure photo of the removed pin from the vermiform appendix is embedded.

Plain abdominal x-ray showed no fluid-air levels, but a metallic foreign body (pin) under right superior pubic bone was apparent [Fig 1]. White blood cells were elevated. Surgical exploration was performed under general anesthesia. Inguinal canal is opened through transverse lower abdominal skin crease. Through swollen cremaster muscle and hernia sac we palpated a sharp metallic foreign body. Sharp side came from appendix lumen, two thirds of pin being in its apex. Dividing cremaster muscle we opened swollen hernia sac and we found the inflamed vermiform appendix perforated by a domestic pin [Fig. 2]. The base of the appendix and coecum were in the internal ring closing it, thus blocking the fluid from the hernia sac returning to the abdominal cavity. Serous-purulent exudate in hernia sac was aspirated.

Appendectomy and high ligation of hernia sac was performed. The wound was primary closed, without drainage. Antibiotics (ceftriaxon 500 mg and gentamicin 40 mg) twice a day for two days intravenously were administered. For postoperative analgesia paracetamol suppositories are given. Patient had uneventful postoperative course, and no complications in three years follow up.

From parents we learned that the boy three weeks before the operation unintentionally ingested a few pins while drinking cola from the glass in a family ceremony.

His mother has removed the pins from his mouth, and since he didn't have any complaints, he wasn't examined regarding foreign bodies in gastro-intestinal tract.

## Discussion

There are several clinical manifestation of Amyand's hernia: reducible or incarcerated hernia within non-inflamed appendix, or inflamed appendix (hernia appendicitis) and ingested foreign body which may be metallic or non metallic in appendix causing perforation or not. Nowadays all these presentations of vermiform appendix within inguinal hernia sac are called Amyand's hernia. Non inflamed appendix in children is found in about 1% of herniotomies, usually as incidental finding. Inflamed vermiform appendix in inguinal hernia sac (hernia appendicitis or Amyand's appendicitis) is ten-folds rarest [4-6].

Foreign body (pin) Amyand's appendicitis is extremely rare, perhaps one case per century. The first published case by Amyand was in London an 11-year-old boy complaining of right inguinal hernia and fistulous abscess. In inguinal hernia sac he found the vermiform appendix and a fistula tract caused by the perforation by ingested pin. Trans-hernia sac appendectomy was done. Half-hour surgery was very painful to the patient and very laborious to surgeon, after one month the patient recovered, but the hernia recurred [7]. Hundred and fifty years later in New York, in 1886 Hall had a similar case of 17-year-old boy (incarcerated Amyand's hernia pin perforated appendicitis) and trans hernia sac appendectomy and herniorrhaphy was done. Patient recovers, but hernia was recurrent. This is the first successful appendectomy recorded in USA [3]. Fowler's review (1912) collected 63 published cases of pins in the appendix, 23 of them in children under eleven years. In this series of cases only four cases have been Amyand's hernias [8]. Watson (1923) collected 512 cases of hernia of the appendix (about 55% of them being in inguinal hernia), and Ryan has collected 537 published cases of vermiform appendix within inguinal hernia up to 1937 [4]. Reviewing of English language surgical literature from 1937 to 2006 on acute appendicitis presenting within an inguinal or femoral hernia Meinke found only eight cases of children and in all of them inflamed appendix vermiform was found in inguinal hernia [9]. Recently no pin hernia appendicitis was reported [10-12][13].

271 years after Amyand, and 120 years after Hall we operated on 6-year-old boy with right incarcerated Amyand's hernia pin perforated appendicitis. Appendectomy and herniotomy was done and patient had uneventful course. During three year follow-up no recurrence occurred.

Historically Amyand's hernia is diagnosed intra-operatively, but preoperative Ultrasound and/or CT scan (2000) can make a correct diagnosis [12,13].

## Conclusion

Foreign body (pin) Amyand's hernia appendicitis seems to be extremely rare, maybe once in a century (Amyand 1735, Hall 1886, and our case in 2006). In patients with clinical signs of incarcerated inguinal hernia, with locally inflammatory signs, but without signs of intestinal obstruction Amyand's hernia appendicitis in differential diagnosis must be considered. Using ultrasound or CT scan correct preoperative diagnosis can be made. In our case, it is possible that the injury during the football game might have induced perforation of the vermiform appendix by the foreign body (domestic pin) in it swallowed three weeks ago.

## Consent

Written informed consent was obtained from the patient's parent for publication of this case report and accompanying images. A copy of the written consent is available for review by the Editor-in-Chief of this journal.

## Competing interests

The authors declare that they have no competing interests.

## Authors' contributions

SL - performed surgery, designed, made literature searching and was a major contributor in writing the manuscript. NH- was a major contributor in designing and writing the manuscript. BK - was major contributor in searching literature and preparing the photos. HJ - has contributed in literature searching and in writing manuscript. AH - has contributed in designing and writing the manuscript. All authors read and approved the final manuscript.
